# Specialists' Perspectives on Social Stories in Managing Individuals With Autism in the Dental Care Setting

**DOI:** 10.1111/scd.70085

**Published:** 2025-08-25

**Authors:** Sobia Zafar, Mim Griffiths, Emily Hartfiel, Andie Malawkin, Claudia Lopez‐Silva

**Affiliations:** ^1^ The University of Queensland Herston Qld Australia

**Keywords:** autism, pediatric dentistry, social stories, special care dentistry

## Abstract

**Aims:**

To investigate and compare the experiences of specialists in pediatric and special care dentistry (SCD) using social stories as a management tool in the dental treatment of individuals on the autism spectrum

**Methods:**

A 34‐item online questionnaire was designed and distributed to specialists in Queensland. Univariate data were analyzed and graphed.

**Results:**

A response rate of 62% was achieved. Results showed that 72% of participants agreed with the statement that “social stories are useful when treating patients with autism.” More respondents reported using paper‐based social stories (44%) than app‐based (33%); however, 61% of participants responded they were more likely to use an app‐based social story when given the choice. There was no statistically significant association between perception of social story usefulness and type of specialty (*p* = 0.627). As autism level of support increased, there was a decrease in perceived benefits of social stories (94% for ASD level 1 vs. 44% for ASD level 3).

**Conclusion:**

This study showed that both Queensland specialists in pediatric dentistry and SCD find social stories to be a useful tool in the management of patients with autism. Further research would enable greater insights into the perception of social stories among dental specialists across Australia.

## 1 Introduction

Autism is a developmental condition characterized by deficits in social communication and interaction skills [1, 2, 3]. According to the Diagnostic and Statistical Manual of Mental Disorders, fifth edition (DSM‐V), the diagnosis of this neurodevelopmental condition includes three support levels; requiring support (Level 1), requiring substantial support (Level 2), and requiring very substantial support (Level 3) [1]. Key features of autism include impaired social development, sensory disturbances and difficulty with everyday planning due to restrictive and repetitive behaviors. According to the DSM‐V, those with autism Level 1 may be able to speak in full sentences but have decreased interest in social communication. An autistic individual Level 3 however may have few words of intelligible speech and rarely initiate social interaction. Individuals with ASD Level 1 may have difficulty with new activities due to inflexibility of behavior, whereas ASD Level 3 individuals have extreme difficulty coping with change with restrictive and repetitive behaviors markedly interfering with function [1].

The etiology of autism is still unclear, but it is considered multifactorial, involving a combination of genetic and nongenetic factors [2]. In 2022, approximately 1.1% Australians were diagnosed with autism according to the Australian Bureau of Statistics (ABS) Survey of Disability, Ageing and Carers (SDAC) [4]. Autism is most prevalent among children aged 5–14, with a prevalence of approximately 4.3% [4]. Autistic children often present with poorer measures of dental health [5, 6]. Specifically, it has been found that this population group may have higher bacterial plaque loads, poorer gingival health, and higher caries rates [5, 6].

Individuals with autism may have high sensitivity to sensory stimuli in areas such as tactile, olfactory, auditory, and visual systems. Coupled with differing communication needs, these factors can impact the dental management of those with autism [3]. For example, the tactile system receives information about light touch, pressure, vibration, temperature, and pain [3]. When individuals are over‐responsive to this sense, their ability to maintain a calm activity level is affected, and as a result, they present as hyper‐alert in dental settings. They can also experience difficulties with oral hygiene, particularly with the texture and taste of toothpaste [3]. This, together with poor visual–motor integration, leads to an increased risk of dental caries and periodontal disease [3]. As such, there is a need for studies that investigate methods that may improve the experience of patients with autism in the dental environment, in order to make dental care more accessible.

Several communication strategies have been suggested for dental clinicians to use when treating young individuals with autism, such as the tell‐show‐do technique [3]. However, this strategy may prove challenging in a noisy dental clinic, especially for those with auditory processing difficulties. Children who are over‐responsive to auditory input experience difficulties functioning when there is a lot of noise around. This may influence their ability to focus and follow instructions within the dental setting. Therefore, other strategies should be implemented in the dental settings as adjunctive measures to assist individuals with communication issues.

These approaches may include picture exchange communication systems and social stories. Social stories are a learning tool created by Carol Gray in the early 1990s to help those with autism better understand social situations and develop desired behaviors [7, 9]. A social story is a short story written and illustrated to describe a particular activity and the behaviors that are expected in that situation [9, 10, 11]. Traditionally, social stories are paper‐based; however, recent developments have allowed social stories to be presented electronically, such as in the form of an application (app). Some studies note that app‐based social stories can be useful for improving the behaviors of autistic patients, as technology may be more easily accessible compared to traditional paper‐based social stories [10, 11, 12, 13]. Furthermore, individuals with autism appear to have a natural affinity for technology [10]. Multiple studies have focused on the effects of social stories in the field of education, showing that they can reduce meltdowns and improve responses to receiving directives [13, 14, 15]. Individuals with autism often adhere to routine, and social stories can provide a rigid structure for the expected what, who, when, where, and why of a social situation [1, 13, 14]. However, there is limited research on the use of paper‐based social stories in the dental context, and no studies have focused on app‐based social stories in the dental setting. From the available literature, social stories can enhance oral hygiene habits, improve behaviors during the dental examination, and potentially reduce perioperative anxiety [15, 16, 17]. Social stories, therefore, may have the potential to aid clinicians in the dental management of patients on the autism spectrum.

The aim of this study was to explore the perceptions and compare the experiences of specialists in SCD (special care dentistry, equivalent to special needs dentistry [SND] within the Australian context) and pediatric dentistry using social stories as a management tool in the treatment of autistic individuals. The objective of this study was to gather data from Queensland specialists using a questionnaire to compare their experiences using social stories and their opinions on using app‐based compared to paper‐based social stories.

## 2 Methodology

### 2.1 Ethical Considerations

Ethics approval was obtained from the Institutional Human Research Ethics Committee (HREC) of The University of Queensland (Approval No. 2023/HE000863).

### 2.2 Survey Tool

The research design was a cross‐sectional questionnaire‐based study with 34 items, surveying specialists in SCD and pediatric dentistry in Queensland, Australia. The questionnaire was designed and delivered using the online program Qualtrics^XM^ (Qualtrics, Provo, Utah, USA). The questionnaire consisted of three categories: (1) Demographics of the participants; (2) role and confidence of participants in treating individuals with autism; and (3) knowledge and prior use of social stories. Category one contained nine items to gather demographic data, including age, gender, years of experience, and type of specialty. Category two contained four items to determine how often clinicians saw patients with autism, which methods they used to treat them, and how confident they were in management. Category three contained 21 items to explore current use of social stories, use of different formats (paper‐ or app‐based), and perceived usefulness of social stories. Most items were presented in multiple‐choice format; some questions also had the ability to select multiple options. Some questions had “other” as an option, where participants could write their own response to better reflect their opinion if necessary. While the study was not formally validated, it was designed and piloted in collaboration with one specialist in pediatric dentistry and one specialist in SCD. Refinements to the questionnaire were made to improve its comprehension for the target specialist audience.

### 2.3 Participant Selection and Recruitment

Inclusion criteria for the study required participants to be registered with the Australian Health Practitioner Regulation Agency (AHPRA) as either specialists in SCD or pediatric dentistry. Participants must work in a dental clinic (private, public, or both) in Queensland, Australia. Participants also must have previously treated autistic patients. Participants were then excluded if they had restrictions on practice scope or limitations on registration that would prevent them from being able to treat patients with autism. Participants from Queensland who were also members of the Australian and New Zealand Academy of Special Needs Dentistry (ANZASND) and the Australian and New Zealand Society of Paediatric Dentistry (ANZSPD) were invited to take part in the study. The survey was distributed via email in March 2024 to be completed within 2 weeks.

### 2.4 Data Analysis

Anonymous responses were transferred onto Microsoft Excel version 2407 (Microsoft Corp., Redmond, Wash., USA). Incomplete responses were excluded from analysis. Univariate analyses were completed using Jamovi (version 2.3.24) and graphed using GraphPad PRISM (GraphPad Software, San Diego, California, USA). Data were presented in graph and table format (numbers and percentages). Fisher's exact test was used to determine if there was a significant association between categorical variables. *p* values of ≤0.05 were considered statistically significant.

## 3 Results

A total of 21 responses were received out of the 29 specialists invited to participate in the study; however, three responses were excluded due to incompletion. The response rate was 62% (18 out of 29). Out of the 24 Queensland specialists in paediatric dentistry, 14 completed the survey (58%). Out of the five specialists in SCD who met the criteria in Queensland, four completed the survey (80%). Of the 18 respondents, 33% were males and 67% were females (Table 1). Age of participants ranged from 31 to 60 years old. Among the respondents, 78% were paediatric dentists, and 22% were specialists in SCD. Years of experience incorporating both general and specialty experience vary, as shown in Table 1. For practice type, six respondents were working in private practice only (33%), five in public (28%), and seven in both private and public practices (39%).

**TABLE 1:** Biographic and demographic information of the participants.

Question	Response	*N* (%)
Gender	Female	12 (67)
	Male	6 (33)
Age range (years)	21—30	0 (0)
	31—40	8 (44)
	41—50	6 (33)
	51—60	4 (22)
	60+	0 (0)
Years of practice as a general dentist (years)	2–4	5 (28)
	5–10	7 (39)
	11+	6 (33)
Type of specialty	Paediatric dentistry	14 (78)
	Special needs dentistry	4 (22)
Years of practice as a specialist (years)	0	1 (5)
	1–4	5 (28)
	5–10	5 (28)
	11+	7 (39)

The data were analyzed to determine the perceived usefulness of social stories in the management of patients with autism, perceived differences in the usefulness of social stories depending on their format (app‐based vs. paper‐based), and perceived differences in the usefulness of social stories based on specialty (paediatric dentistry vs. SCD). These variables were explained by the specialty of the participant (pediatric dentistry or SCD) and the age of the patient with autism (adult vs. child). The possible covariates considered during the analysis were years of practice as a specialist, prior experience and confidence treating patients with autism, knowledge and prior use of social stories, and the level of autism functioning of patients.

Over half of respondents (56%) care for autistic patients daily (Table 2). All respondents (100%) use non‐pharmacological behavioral management techniques such as tell‐show‐do, distraction, and positive reinforcement. Participants report using paper‐ and app‐based social stories, as well as other non‐pharmacological behavioral management techniques such as sensory toys, modelling, play‐based interventions, and discussions based on patient interests. Majority of participants reported they felt very confident in managing and treating patients on the autism spectrum (67%), as shown in Table 2.

**TABLE 2:** Role and confidence of paediatric dentists and special care dentists in managing autistic patients.

Question	Response	*N* (%)
On average, how often do you treat autistic patients?	Daily	10 (56)
	Weekly	5 (28)
	Fortnightly	1 (6)
	Monthly	0 (0)
	Yearly	2 (11)
	Less than yearly	0 (0)
Do you use any non‐pharmacological behavioural management techniques?	Yes	18 (100)
	No	0 (0)
	Unsure	0 (0)
How do you rate yourself at managing and treating autistic patients?	Very confident	12 (67)
	Somewhat confident	6 (33)
	Neutral	0 (0)
	Not very confident	0 (0)
	Avoid providing dental treatment to patients on the autism spectrum	0 (0)
Which of these tools/methods do you use? (select all that apply)	Paper‐based social stories	7 (39)
	App‐based social stories	7 (39)
	Tell‐show‐do	18 (100)
	Distraction	18 (100)
	Positive reinforcement	18 (100)
	Other	4 (22)

With regards to knowledge and prior use of social stories, 83% of respondents stated that they are aware of what a social story is, and 17% were unsure. Over half of the clinicians surveyed (56%) had used a paper‐based social story previously, with 44% using it in their current practice. In contrast, 39% had used an app‐based social story previously, with 33% reporting current use.

**FIGURE 1:**
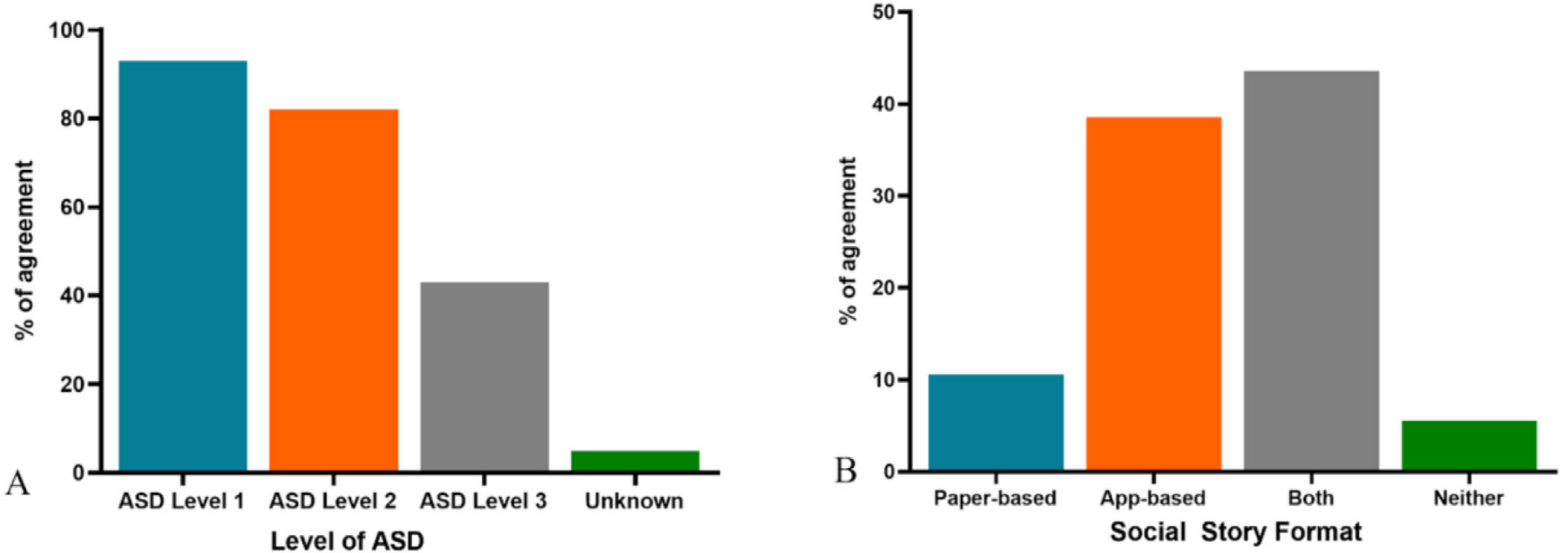
(A) Percentage of participants who found social stories beneficial for different levels of support required by autistic patients; (B) percentage of social story format preference.

Results showed that 72% of participants agreed with the statement that social stories are very useful or somewhat useful when treating patients on the autism spectrum. The remaining 28% felt neutral. Clinicians were more likely to use social stories as a dental management tool for children on the autism spectrum compared to autistic adults (83% vs. 72%). As the level of support increased for these individuals, there was a decrease in the perceived benefits of social stories. Some 94% of respondents found social stories useful for patients with ASD level 1, compared to 83% for ASD level 2 and 44% for ASD level 3 (Figure 1A).

Comparing the usefulness of different social story formats, 77% of participants find paper‐based social stories very or somewhat useful, compared to 55% for app‐based social stories. All respondents who had used a paper‐based social story previously (56%) noticed improved compliance during dental treatment of autistic patients. Similarly, all respondents who had used app‐based social stories before (39%) reported improved compliance.

In general, most participants responded that both paper‐ and app‐based social stories should be more commonly used as a dental management tool when treating patients on the autism spectrum (67% for paper‐based and 61% for app‐based). When participants were asked whether they were more likely to use paper‐ or app‐based social stories, 61% responded app, compared to 17% for paper. In a similar question, participants were asked whether they would rather use paper‐ or app‐based social stories: 44% responded both, 39% responded app, and 11% responded paper alone (Figure 1B).

There was no statistically significant association between type of specialty and current use of paper‐ and app‐based social stories (*p* = 1.000, *p* = 0.569). There was no statistically significant association between type of specialty and preference of social story format (*p* = 0.808), or likelihood to use a certain format (*p* = 0.808). There was no statistically significant association between the type of specialty and perception of social story usefulness (*p* = 0.627).

**FIGURE 2:**
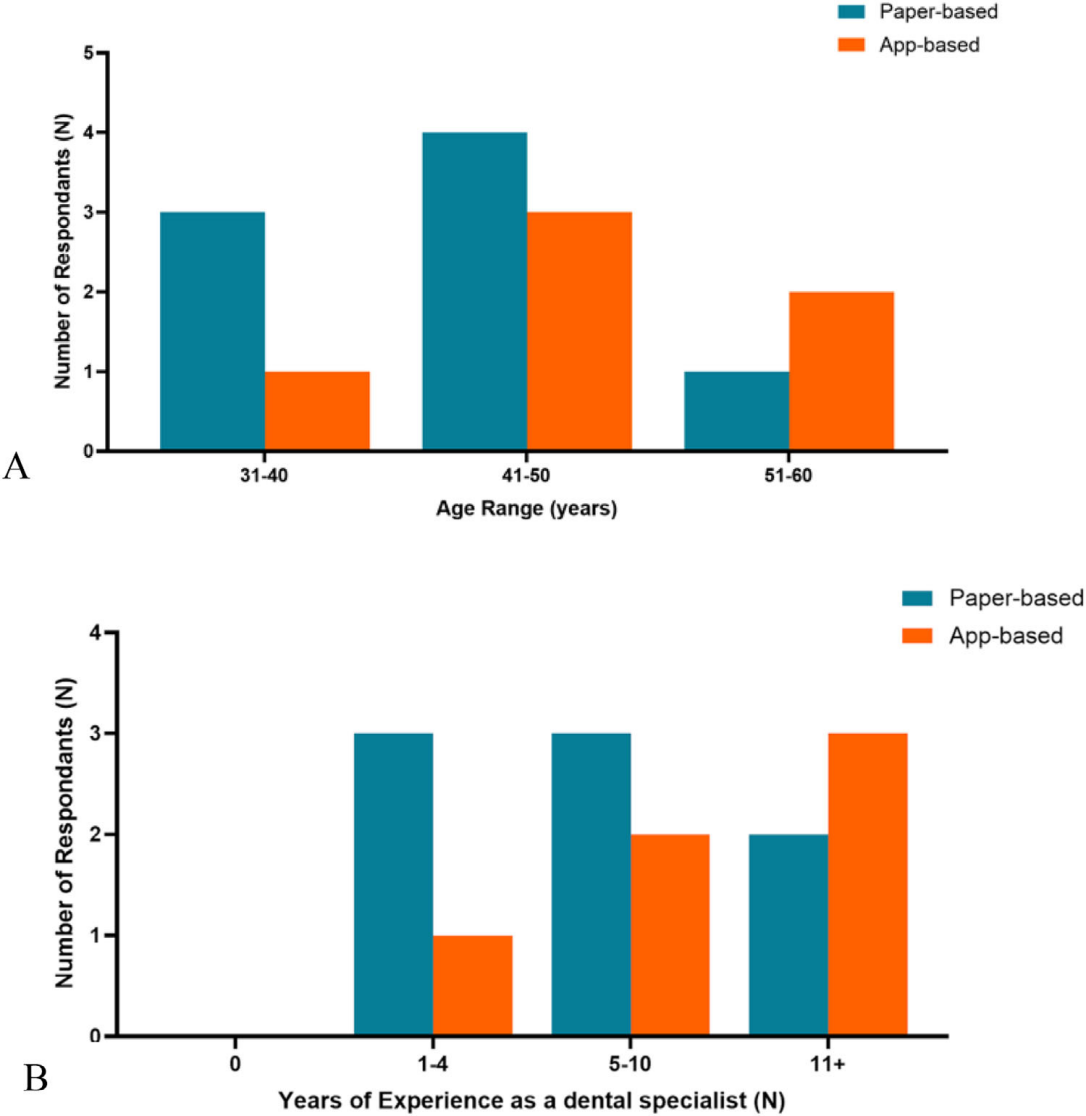
(A) Age of participants versus social story format current usage; (B) participants' years of experience versus social story format.

There was no statistically significant association between the age of the specialist and current use of paper‐based social stories (*p* = 0.574), current use of app‐based social stories (*p* = 0.247), or preference between the two social story formats (*p* = 0.260) (Figure 2). Additionally, years of practice as a specialist did not have a statistically significant association with current use of paper‐ or app‐based social stories (*p* = 0.887, *p* = 0.680).

## 4 Discussion

The available literature generally agrees that social stories are an effective method for creating behavioral changes in autistic people [9, 11]. However, the inherent variability within social stories, how they are delivered, and the diversity within autistic people creates variability within the results [9, 11]. As such, in their scoping review, Camilleri et al. suggest that social validity should be considered when assessing the effectiveness of interventions [11]. Social validity refers to the perceived effectiveness of the intervention by the service users [11]. In the case of social stories, this would include autistic people, as well as their parents, caregivers, educators, and healthcare workers. Ali et al. advocated for a similar idea, that the definition of “successful outcomes” should be expanded to include those working with autistic people, as well as the subjects themselves [1]. Camilleri et al. reported that when assessing social validity, social stories were found to be a very effective tool in changing behaviors of autistic people [11].

In their randomized control trial, Wright et al. also used social validity as a measure of effectiveness for social stories as a behavioral intervention in UK primary schools [19]. While they did not find statistically significant differences between intervention and control groups using standardized behavioural scales (Social Responsiveness Scale 2), they did find that children receiving the social story intervention were statistically more likely to achieve socio‐emotional goals set by the children in collaboration with their teachers and parents [19]. They also report that parents and teachers find social stories to be a useful and low‐cost behavioral intervention for children with autism [19].

The current study measured the social validity of social stories among dental specialists treating patients with autism. Queensland specialists in SCD and paediatric dentistry report that social stories are helpful in the dental treatment of patients on the autism spectrum, with the vast majority reporting improved compliance after use, and finding them useful overall. This is corroborated by existing research in other fields such as medicine and education, which found that social stories can reduce meltdowns, improve the capacity to follow instructions, and decrease perioperative anxiety [13, 14, 16]. One possible explanation for this is that social stories communicate the desired behaviors in a convenient and unobtrusive manner, which focuses on the strengths exhibited by individuals with autism [14, 17]. Despite the majority of respondents finding social stories useful, less than half of the respondents reported current use of social stories in any format.

When comparing paper‐ and app‐based social stories, more specialists responded that they find paper‐based useful compared to app‐based. This may be explained by the greater number of current and prior use of paper‐based social stories. Despite this, when given the option, specialists were more likely to choose the app over paper‐based social stories. Research in other fields shows promising data regarding app‐based social stories compared to traditional paper‐based social stories, with improved accessibility and personalization [12]. Studies show that individuals with autism may have a greater affinity for technology and find app‐based social stories to be more engaging due to their customizability [20]. App‐based social stories can also reduce environmental footprint and increase accessibility because individuals can have multiple social stories on one portable device for different situations, rather than printing and carrying around multiple stories.

The lack of current use of social stories may indicate potential barriers faced by specialists attempting to implement them in their practice. Several participants responded that their use of social stories, or their choice of app versus paper, is largely dependent on the individual patient. For example, each patient may have their own previous experience or preference for a particular social story format and may benefit from maintaining their routine. Individuals with autism may have differences in processing stimuli and favor planning out the steps of activities, and therefore may display ritualized or rigid behavior patterns [2]. As such, routine provided by a social story can be helpful for these individuals, as it offers structure and can minimize aversiveness to change [21].

For some patients, social stories may not be a useful tool. Specialists responded that social stories are more beneficial for autistic individuals who require lower levels of support (Level 1), and less beneficial for those who require higher levels of support (Level 3). This is likely because it is easier for individuals on the autism spectrum Level 1 to engage in communication, and they are less inflexible to change compared to individuals on the autism spectrum Level 3 [1]. As such, they may benefit from having someone read them a social story or navigating one themselves, as it may prepare them for an upcoming situation.

There were limitations in the research involving the study design and data collection. To avoid nonresponse bias, the questionnaire was designed to be as short as possible and digital so that it was easily accessible. This may have prevented nonresponse; however, only a 62% response rate was achieved overall. The moderate sample size may impact the generalizability of the findings from this study in the Australian and international context; however, the focus of this study was to acquire information regarding specialist experience in using social stories in Queensland without expecting to generalize the results to other states or countries. A quantitative study was designed to minimize the time burden placed on specialist dentists, as quantitative surveys are simpler and quicker to complete for participants compared to qualitative study designs [22]. The current study could provide fundamental information to develop questions for a subsequent qualitative study to explore deeper insights into this topic.

This study demonstrates that most Queensland specialists in SCD and paediatric dentistry find social stories to be a useful tool in the treatment of patients on the autism spectrum. Clinicians may benefit from using social stories in their own practices to improve compliance of autistic patients. Further research could explore barriers and facilitators to implementing social stories clinically and work to expand the use of social stories in dental practices.

## 5 Conclusion

This study showed that Queensland specialists in both SCD and paediatric dentistry find social stories to be a useful tool in the management of patients on the autism spectrum. A significant proportion of Queensland specialists in SCD and Paediatric dentistry were surveyed in accordance with the objective of this study. Despite less historical use, clinicians have positive attitudes toward app‐based social stories, which may allow personalization for individualized treatment and may be more accessible for patients and caregivers. While the results of this study may be limited by collecting data quantitatively rather than qualitatively, it sheds light on the importance of future research into the perception of social stories among dental specialists across Australia. This may help to identify barriers and enablers in the implementation of app‐based social stories to facilitate communication in dental practices.

## Author Contributions

Sobia Zafar and Claudia Lopez‐Silva conceived the ideas; Mim Griffiths, Emily Hartfiel, and Andie Malawkin wrote the methodology; collected the data; analyzed the data; and contributed to the writing. Sobia Zafar and Claudia Lopez‐Silva supervised, analyzed the data, and edited the manuscript.

## Supporting information




**Supporting Table 1**: Questionairre distributed to participants.
