# Supplementary material for: Specialists' Perspectives on Social Stories in Managing Individuals With Autism in the Dental Care Setting
Source: Spec Care Dentist. 2025 Aug 25;45(4):e70085. doi: 10.1111/scd.70085 (PMC12379069; doi:10.1111/scd.70085)
Supplement: Supplementary file 1 — Supporting Table 1: Questionairre distributed to participants. [file SCD-45-0-s001.docx]

**Table S1: Questionnaire distributed to participants**

**SECTION A - BIOGRAPHIC AND DEMOGRAPHIC INFORMATION**

1. What gender do you identify as?

- Female
- Male
- Non-binary
- Prefer not to say

2. What is your age range (years)?

- 21-30
- 31-40
- 41-50
- 51-60
- 60+

3. How many years did you practice as a General Dentist?

- 2-4
- 5-10
- 11+

4. What kind of dental specialist are you?

- Paediatric Dentist
- Special Needs Dentist

5. How many years have you been practicing as a Specialist?

- 0
- 1-4
- 5-10
- 11+

6. For your specialty training, which country did you graduate from?

- Australia
- New Zealand
- Other (please specify): ____________________________________________

7. For your speciality training, which university did you graduate from?

- The University of Queensland
- University of Sydney
- University of Melbourne
- University of Western Australia
- University of Otago
- Other (please specify):______________________________________________

8. What year did you graduate from your specialty?

- Before 1970
- 1971-1980
- 1981-1990
- 1991-2000
- 2001-2010
- 2011-2023

9. Which kind of practice do you work in?

- Private
- Public
- Both

**SECTION B - ROLE AND CONFIDENCE IN MANAGING PATIENTS WITH ASD:** This section is designed to ask you about your experiences with using social stories and treatment of patients with ASD.

10. On average, how often do you treat patients with ASD?

- Daily
- Weekly
- Fortnightly
- Monthly
- Yearly
- Less than yearly

11. Do you use any non-pharmacological behavioural management techniques?

- Yes
- No
- Unsure

12. How do you rate yourself at managing and treating patients with ASD?

- Very confident
- Somewhat confident
- Neutral
- Not very confident
- Avoid providing dental treatment to patients with ASD

13. Which of these tools/methods do you use? (Please select all which apply)

- Paper-based social stories
- App-based social stories
- Tell-show-do technique
- Distraction technique
- Positive reinforcement
- Other (please specify):__________________________________________________

**SECTION C - KNOWLEDGE AND PRIOR USE OF SOCIAL STORIES**

14. Do you know what Social Stories are?

- Yes
- No
- Unsure

15. How would you rate your experience using social stories in the dental management of patients with ASD?

- Very useful
- Somewhat useful
- Neutral
- Somewhat unuseful
- Not useful

16. Do you think social stories are beneficial in patients with the following levels of ASD? (Select all which apply)

- ASD Level 1
- ASD Level 2
- ASD Level 3
- Unknown

17. Would you use social stories as a dental management tool for a child with ASD?

- Yes
- No
- Unsure

18. Would you use social stories as a dental management tool for an adult with ASD?

- Yes
- No
- Unsure

19. Have you used a paper-based social story before?

- Yes
- No
- Unsure

20. Do you use a paper-based social story at your current practice?

- Yes
- No
- Unsure

21. How likely would you be to use a paper-based social story?

- Very likely to
- Somewhat likely to
- Neutral
- Somewhat unlikely to
- Not likely to

22. Rate how useful you think paper-based social stories are in patients with ASD

| Very useful | Somewhat useful | Neutral | Somewhat unuseful | Very unuseful |
| --- | --- | --- | --- | --- |

23. Have you noticed improved compliance in patients who have used a paper-based social story before dental treatment?

- Yes
- No
- Unsure
- Have not used a paper-based social story
- Other (please specify):______________________________________________

24. Do you think paper-based social stories should become more commonly used in dental management of patients with ASD?

- Yes
- No
- Unsure
- Other (please specify):______________________________________________

25. Have you used an app-based social story before?

- Yes
- No
- Unsure

26. Do you use an app-based social story at your current practice?

- Yes
- No
- Unsure

27. How likely would you be to use an app-based social story?

- Very likely to
- Somewhat likely to
- Neutral
- Somewhat unlikely to
- Not likely to

28. Have you noticed improved compliance in patients who have used an app-based social story before dental treatment?

- Yes
- No
- Unsure
- Have not used an app-based social story
- Other (please specify):______________________________________________

29. Do you think app-based social stories should become more commonly used in dental management of patients with ASD?

- Yes
- **No**
- Unsure
- Other (please specify):______________________________________________

30. Rate how useful you think app-based social stories are in patients with ASD

| Very useful | Somewhat useful | Neutral | Somewhat unuseful | Very unuseful |
| --- | --- | --- | --- | --- |

31. Are you more likely to use an app or paper based social story?

- App-based
- Paper-based
- Neither
- No preference

32. Do you think app-based social stories are more beneficial compared to paper-based social stories?

- Yes
- No
- Unsure
- Other (please specify):______________________________________________

33. Would you rather use an app-based or paper-based social story in the dental management of a patient with ASD?

- App-based
- Paper-based
- Neither
- Both

34. Any other comments: _______________________________________________________________________________________________________________________________________________________________________________________________________________________________________________________________________________________________________________________________________________________________________________________
